# Diversity and geographic distribution of soil streptomycetes with antagonistic potential against actinomycetoma-causing *Streptomyces sudanensis* in Sudan and South Sudan

**DOI:** 10.1186/s12866-020-1717-y

**Published:** 2020-02-12

**Authors:** Mohamed E. Hamid, Thomas Reitz, Martin R. P. Joseph, Kerstin Hommel, Adil Mahgoub, Mogahid M. Elhassan, François Buscot, Mika Tarkka

**Affiliations:** 1 Department of Soil Ecology, Helmholtz-Centre for Environmental Research GmbH – UFZ, Theodor-Lieser-Str. 4, 06120 Halle, Germany; 2 Department of Clinical Microbiology and Parasitology/ College of Medicine, King Khalid University, PO Box 641, Abha, 61314 Saudi Arabia; 3 Department of Preventive Medicine, Faculty of Veterinary Science, University of Khartoum, Khartoum, Sudan; 4 German Centre of Integrative Biodiversity Research (iDiv), Halle – Jena – Leipzig, Germany; 5 Department of Clinical Laboratory Science, College of Applied Medical Science, Taibah University, Medina, Saudi Arabia

**Keywords:** Streptomycetes, Actinomycetoma, Soil microbiome, In vitro analysis, Phenotyping, Antagonistic potential, *S. sudanensis*, 16S rRNA gene, Sudan

## Abstract

**Background:**

Production of antibiotics to inhibit competitors affects soil microbial community composition and contributes to disease suppression. In this work, we characterized whether *Streptomyces* bacteria, prolific antibiotics producers, inhibit a soil borne human pathogenic microorganism, *Streptomyces sudanensis. S. sudanensis* represents the major causal agent of actinomycetoma – a largely under-studied and dreadful subcutaneous disease of humans in the tropics and subtropics. The objective of this study was to evaluate the in vitro *S. sudanensis* inhibitory potential of soil streptomycetes isolated from different sites in Sudan, including areas with frequent (mycetoma belt) and rare actinomycetoma cases of illness.

**Results:**

Using selective media, 173 *Streptomyces* isolates were recovered from 17 sites representing three ecoregions and different vegetation and ecological subdivisions in Sudan. In total, 115 strains of the 173 (66.5%) displayed antagonism against *S. sudanensis* with different levels of inhibition. Strains isolated from the South Saharan steppe and woodlands ecoregion (Northern Sudan) exhibited higher inhibitory potential than those strains isolated from the East Sudanian savanna ecoregion located in the south and southeastern Sudan, or the strains isolated from the Sahelian Acacia savanna ecoregion located in central and western Sudan. According to 16S rRNA gene sequence analysis, isolates were predominantly related to *Streptomyces werraensis*, *S. enissocaesilis*, *S. griseostramineus* and *S. prasinosporus*. Three clusters of isolates were related to strains that have previously been isolated from human and animal actinomycetoma cases: SD524 (*Streptomyces* sp. subclade 6), SD528 (*Streptomyces griseostramineus*) and SD552 (*Streptomyces werraensis*).

**Conclusion:**

The in vitro inhibitory potential against *S. sudanensis* was proven for more than half of the soil streptomycetes isolates in this study and this potential may contribute to suppressing the abundance and virulence of *S. sudanensis*. The streptomycetes isolated from the mycetoma free South Saharan steppe ecoregion show the highest average inhibitory potential. Further analyses suggest that mainly soil properties and rainfall modulate the structure and function of *Streptomyces* species, including their antagonistic activity against *S. sudanensis.*

## Background

*Streptomycetes* are high G + C Gram-positive, spore-forming bacteria of the family *Streptomycetaceae* (order Actinomycetales), which includes more than 500 species [1]. They are widely distributed in soils and may exceed in abundance the other soil bacterial genera [2]. Many pathogenic streptomycetes have been isolated from soil samples. A few species are causal agents of diseases in animals: (*S. cyaneus* and *Streptomyces* sp.) [3, 4] and plants such as *S. scabies, S. turgidiscabies, S. luridiscabiei, S. puniciscabiei* and *S. niveiscabiei* [5–7]. Relevant to humans, two species of soil *Streptomyces*, *S. sudanensis* and *S. somaliensis* cause actinomycetoma [8, 9]. As a result of an isolation campaign for medically important actinomycetes from Iranian soils, the human pathogen *Streptomyces somaliensis* was among the most frequently isolated species, representing about 20% of the obtained isolates [8]. This suggests that soils are reservoirs for pathogenic streptomycetes and their propagules. The main vectors for transmission to humans are the long, sharp thorns of Acacia trees. Acacia comes in contact with *S. sudanensis* spores and mycelium probably on the soil surface and the transmission of the bacterium to a human host occurs when people walking barefoot step on an infested thorn, strong enough to puncture the human skin and to deliver the bacterium to the host. Acacia trees grow on a significant proportion in Sudan and South Sudan including the mycetoma belt [10]. This suggests the occurrence of soil-borne infections [11] rather than Acacia risk exclusively. For both thorn prick and soil mediated infections *S. somaliensis* inhibitory soil bacteria may lower the abundance of disease causing streptomycetes and suppress human infection rate.

A global survey of streptomycetes indicated that apart from the impact of environmental filtering, variation in *Streptomyces* inhibitory phenotypes at different geographic locations may also be a consequence of local selection mediated by species interactions [12]. This suggests that an analysis of *Streptomyces* strain collections from different sites is advisable when strong inhibitors of certain organisms are searched for. The presence of human pathogenic streptomycetes in soils and the inhibitory interaction potential of other *Streptomyces* strains led us to evaluate the relative abundances of *S. sudanensis* inhibiting streptomycetes in soils from inside and outside the Sudanian actinomycetoma belt. Assuming that site-specific parameters, like soil type, soil nutrient levels, precipitation, and temperature influence soil microbial diversity and activity, we first hypothesized that these site-specific parameters affect the structure and *S. sudanensis* inhibitory potential of the soil *Streptomyces* community. Since Davelos et al. [13] reported a positive correlation between antibiotic activity and soil density of streptomycetes, we further hypothesized that increased abundance of *Streptomyces* isolates goes along with an increased inhibitory potential against *S. sudanensis*. With our work, we aimed to map the potential of soil streptomycetes to suppress *S. sudanensis* and consequently actinomycetoma cases of disease in Sudan. We further aimed to identify soil parameters and environmental conditions at which *S. sudanensis* inhibiting streptomycetes are enriched.

## Results

### Soil properties and potential soil enzyme activities

Nutrient levels were highly variable among the sites and their corresponding soil types (Table 1). Soil type, land use, nutrient level and pH did not influence microbial enzyme activities in the soils. Instead, annual precipitation amounts were positively related to the activities of microbial enzymes, like β-glucosidases (*p* = 0.0014), cellobiohydrolases (*p* = 0.012), xylanases (*p* = 0.0036) and acid phosphatases (*p* = 0.0025) (Additional files 1 and 2).

**Table 1:** Characterization of sampling sites and *Streptomyces* collections. Climatic conditions and soil properties at the sampling sites, microbial enzyme activities, the numbers of isolates recovered from each site along with their average antagonism against *Streptomyces sudanensis* are listed

Site code	Site name (coordinates)	Ecoregion^a^	Soil type^b^ (Common description)	Average annual temperature (°C)	Average annual rain (mm)	P (mg/100 g)	K (mg/100 g)	Soil pH	NAG Activity (nmol/ hr.^−1^ g soil^− 1^)^c^	GLU Activity (nmol/ hr.^− 1^ g soil^− 1^)^c^	CEL Activity (nmol/ hr.^− 1^ g soil^− 1^)^c^	PHO Activity (nmol/ hr.^− 1^ g soil^− 1^)^c^	XYL Activity (nmol/ hr.^− 1^ g soil^− 1^)^c^	Humic acid: 10^5^ CFU/g soil^− 1^	ISP2:10^5^ CFU/g soil^− 1^	Nr of selected phenotypes	Average antagonism±SE (clearance zone/ colony diameter)
1	Juba (4.8594° N; 31.571° E)	East Sudanian savanna	Nitosols (Tropical rusty red)	28.5	954	1.6	14.6	7.7	9.2	53.6	1.4	37.4	2.9	15	26.1	10	1.46 ± 0.45
2	Hajj Abd Allah (13.58° N; 33.35° E)	Sahelian Acacia savanna	Fluvisols (Riverside silt)	28.3	427	4.5	46.3	8.0	18.9	59.9	3.2	45.6	8.1	8.7	16.8	4	1.62 ± 0.15
4	Hajj Abd Allah (13.58° N; 33.35° E)	Sahelian Acacia savanna	Vertisols (Black clay-Gazira cotton clay)	28.3	427	6.3	28.2	8.1	9.2	50.2	1.7	60.6	5.4	3	15.9	19	1.62 ± 0.35
5	Hajj Abd Allah (13.58° N; 33.35° E)	Sahelian Acacia savanna	Fluvisols (Silt, gorair)	28.3	427	25.3	80.5	8.1	10.7	93.9	2.8	45.1	6.8	6.3	15	11	0.81 ± 0.34
7	Hussein Narti-1 (18.1510° N; 31.1402° E)	South Saharan steppe and woodlands	Yermosol (Desert sand)	29.7	70	4.6	32.6	8.0	2.2	4.1	< 0.05	1.9	0.2	27.6	5.1	15	3.03 ± 0.53
8	El Muglad (11.0347° N; 27.7491° E)	East Sudanian savanna	Arenosols (Stabilized sand dunes with silt or clay)	28.5	501	2.0	28.2	6.4	25.8	54.3	5.0	85.0	8.4	18.3	31.8	16	1.82 ± 0.37
10	Nyala (12.0518° N 24.8805° E)	Sahelian Acacia savanna	Arenosols (Stabilized sand dunes with silt or clay)	27.2	398	1.5	19.2	7.1	9.5	19.3	< 0.05	27.0	1.9	1.8	10.8	4	0.30 ± 0.3
11	Khartoum- Soba west (15.5007° N 32.5599° E)	Sahelian Acacia savanna	Yermosols (Flat gravel silt loam with clay)	29.6	164	8.3	19.1	8.5	3.4	8.8	0.5	6.3	1.6	20.4	27.3	22	2.83 ± 0.37
12	Hajj Abd Allah (13.58° N; 33.35° E)	Sahelian Acacia savanna	Fluvisols (Fine sands)	28.3	427	9.1	59.1	8.0	10.4	69.0	1.3	11.7	4.1	12	22.8	15	2.60 ± 0.46
13	Kassala (15.4581° N 36.4040° E)	East Sudanian savanna	Fluvisols (Riverside silt)	29.6	251	9.0	35.2	7.8	12.3	42.1	0.7	27.2	2.4	7.8	21.3	10	0.64 ± 0.33
14	Umm Ruwaba (12.9028° N; 31.2283° E)	Sahelian Acacia savanna	Arenosols (Stabilized sand dunes with silt or clay)	27.0	375	6.7	22.2	6.8	0.7	9.9	< 0.05	24.0	0.2	6.6	12	11	0.95 ± 0.41
16	Sennar (13.4580° N; 33.2588° E)	Sahelian Acacia savanna	Vertisols (Black clay)	28.3	427	10.8	48.3	8.1	2.4	22.3	0.2	40.4	1.8	4.2	13.2	5	0.00 ± 0.00
19	Hussein Narti-2 (18.1510° N; 31.1402° E)	South Saharan steppe and woodlands	Yermosols (Desert sand)	29.7	70	5.3	38.3	8.0	2.4	4.9	< 0.05	< 0.05	0.2	19.8	26.4	12	2.55 ± 0.60
21	AL Gadarif-Basonda (14.0243° N; 35.3686° E)	East Sudanian savanna	Vertisols (Black clay)	28.9	604	2.4	37.4	7.6	1.9	9.6	1.1	46.1	2.5	2.7	9.9	3	1.11 ± 1.11
23	Al Fashir (13.6198° N; 25.3549° E)	Sahelian Acacia savanna	Arenosols (Stabilized sand dunes with silt or clay)	26.0	213	28.6	60.0	7.7	4.0	12.1	0.5	13.6	1.2	1.8	10.8	2	2.47 ± 0.87
27	Ad Douiem (13.9951° N; 32.2994° E)	Sahelian Acacia savanna	Yermosols (Flat gravel silt loam with clay)	28.3	427	8.9	52.6	8.0	4.0	40.8	0.4	29.2	3.1	16.8	22.5	12	2.91 ± 0.36
29	Ad Damazin (11.7855° N; 34.3421° E)	East Sudanian savanna	Vertisols (Black clay)	28.3	713	16.5	49.1	7.3	18.9	87.2	3.4	80.5	8.7	5.4	19.8	4	1.76 ± 0.61

^a^Terrestrial ecoregions of Africa and Madagascar: a conservation assessment [14]; ^b^Soil typing based on the soil map of Sudan [15]. ^c^Soil hydrolytic enzyme activities: *NAG* N-acetylglucosaminidase, *GLU* Beta-glucosidase, *CEL* Cellulases, *PHO* Phosphatases, *XYl* Xylosidases

### Selective isolation of streptomycetes from soil

Isolation of *Streptomyces* spp. from soil samples was conducted on HA and ISP2 media (Table 1 and Additional file 3). The average numbers of isolates on HA and ISP2 media (CFU × 10^5^/g soil) were in Arenosol 6.3–25, Yermosol 16.4–23.9, Nitosol 20.6 (one sample), Fluvisol 10.7–17, and Vertisol 6.3–12.6. Lowest numbers of isolates were obtained from Yermosol (Additional file 4). Regarding the ecoregions, highest average numbers of isolates were obtained from East Sudanian savanna Arenosol (25.1 × 10^5^) and lowest from Sahelian Acacia savanna Arenosol (6.3) (Table 1).

### Phylogenetic classification of bacteria based on partial 16S rRNA gene

**Fig. 1:**
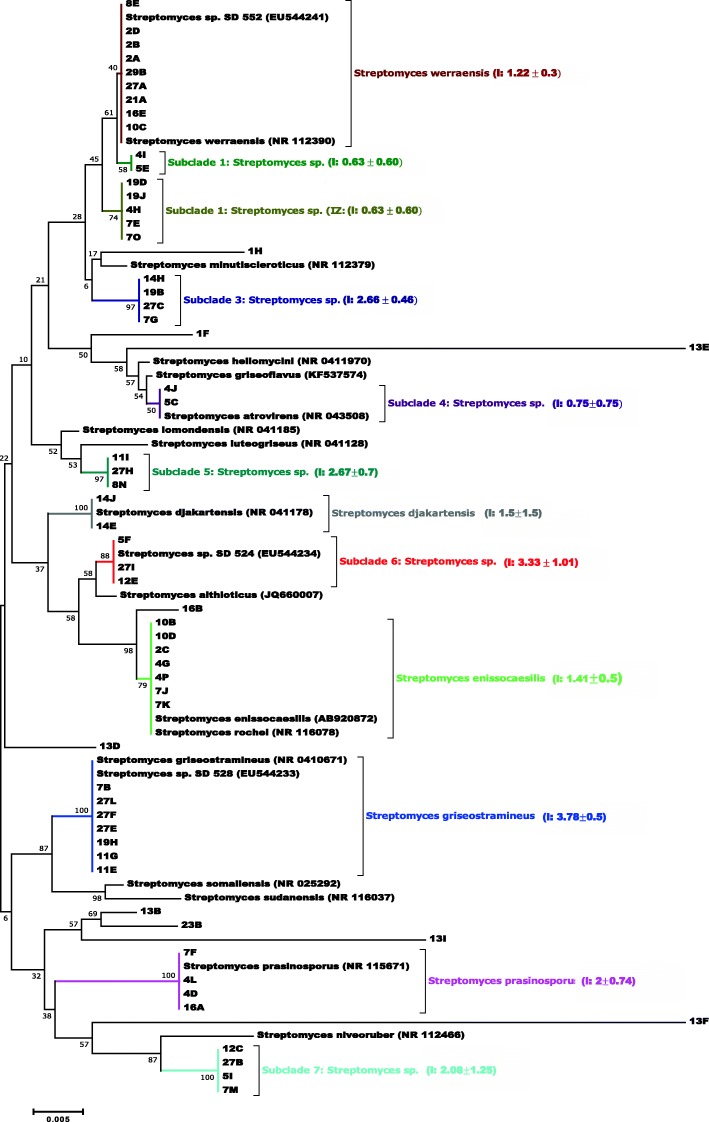
Neighbor-joining tree based on 16S rRNA gene sequences showing relationships of soil streptomycetes with related, validly described *Streptomyces* species (accession numbers in brackets). In vitro inhibition against *S. sudanensis*, i.e.ratios between inhibition zone and colony diameter, are marked as I: Mean value and standard error. Evolutionary analysis was performed using MEGA7 software [16]

The isolates were initially selected by colony morphology and their assignment to the genus *Streptomyces* was confirmed using 16S rDNA sequence analysis. According to partial 16S rRNA gene sequencing (Additional file 4), 173 of 175 isolates were confirmed as *Streptomyces* spp., whereas two strains (13F, 27 K) were *Amycolatopsis* spp. (data not shown). Nucleotide sequence data have been deposited in GenBank and corresponding accession numbers are listed in. Isolate sequences were compared with sequences of *Streptomyces* type strains, and the relationships between the sequences of representative isolates for each cluster and their closest phylogenetic neighbors are shown in Fig. 1. Some sequences formed distinct phylogenetic lines, while others were grouped in clusters in the *Streptomyces* 16S rRNA gene tree. Main subclades identified were: *Streptomyces werraensis* (*n* = 9)*; S. enissocaesilis* (*n* = 7); *S. griseostramineus* (n = 7) and *S. prasinosporus* (*n* = 4). Some strains, grouped in subclades 1, 2, 3, 4, 5, 6 and 7, formed *Streptomyces* clusters that are clearly separated from described species. The phylogenetic positions of these novel clusters were distinguished from one another and from the nearby *Streptomyces* spp. on the basis of 16S rRNA gene sequence similarities (Fig. 1).

The 16S rRNA gene sequences of some isolated soil streptomycetes aligned with *Streptomyces* strains isolated previously from human (SD524, SD528) and animal (SD552) actinomycetoma cases in Sudan (unpublished data). Soil isolates: 2A, 2B, 2D, 8E, 10C, 16E, 21A, 27A and 29B aligned with *S. werraensis* along with *Streptomyces* sp. SD552 (accession EU544241). *Streptomyces* sp. SD524 (accession EU544234) has sequence similarity with strains in subclade 6 (isolates 5F, 12E, 27I). Soil isolates 7B, 11E, 11G, 19H, 27E, 27F, 27 L aligned with *Streptomyces griseostramineus* along with *Streptomyces* sp. SD528 (EU544233).

### The distribution of streptomycetes in Sudanese soils and their antagonistic potential against *Streptomyces sudanensis*

**Fig. 2:**
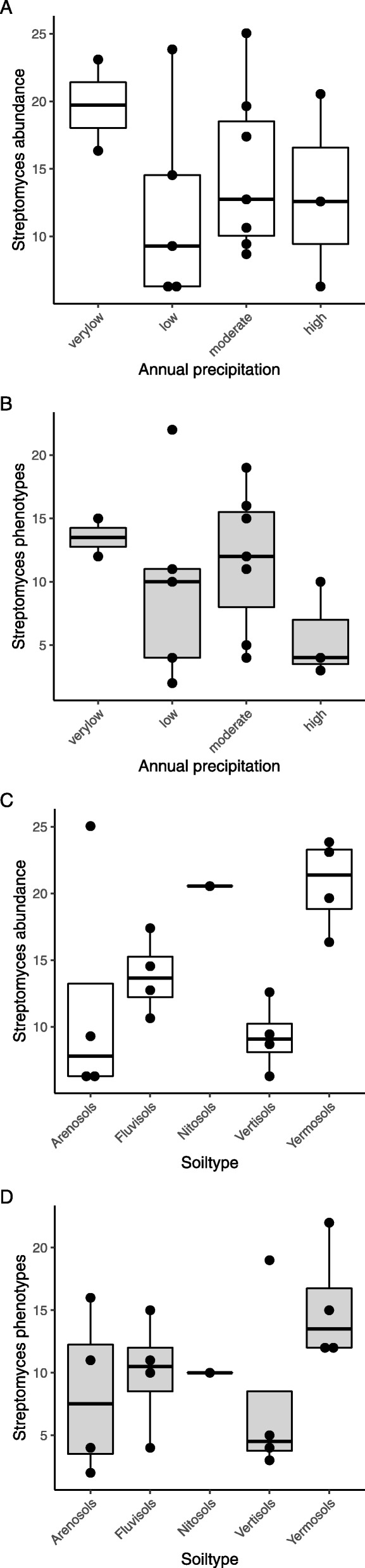
Abundance of streptomycetes and their phenotypic diversity related to precipitation level and soil type. Streptomycete abundance (**a**, **c**) at the level of mean value of colony forming units (× 10^5^ / g soil) on humic acid agar and ISP2 agar, and *Streptomyces* phenotypic diversity (**b**, **c**) as related to annual precipitation and soil type, respectively. Precipitation levels 0–100 mm (very low), 101–400 mm (low), 401–600 mm (moderate) and 601–1000 mm (high). Streptomycete abundance was different between Arenosols and Yermosols (*p* = 0.048) and Vertisols and Yermosols (*p* = 0.012) according to one way ANOVA and Tukey test

Low precipitation favored the abundance and phenotypic diversity of *Streptomyces* species (Fig. 2; Additional file 4 and Additional file 5). Soils from areas with low annual precipitation, 70–200 mm per year (sites 7, 19), showed more *Streptomyces* colonies on humic acid agar *(p* = 0.039) than sites with higher precipitation (Additional file 5). Multiple soil types occur within most of the ecoregions (Table 1). Only in the ecoregion South Saharan steppe and woodlands which has a very low annual precipitation both sampling sites (7 and 19) were located on Yermosols. In these Yermosol soils, a higher abundance of streptomycetes was observed compared to that of Arenosols (*p* = 0.048) and Vertisols (*p* = 0.012).

**Fig. 3:**
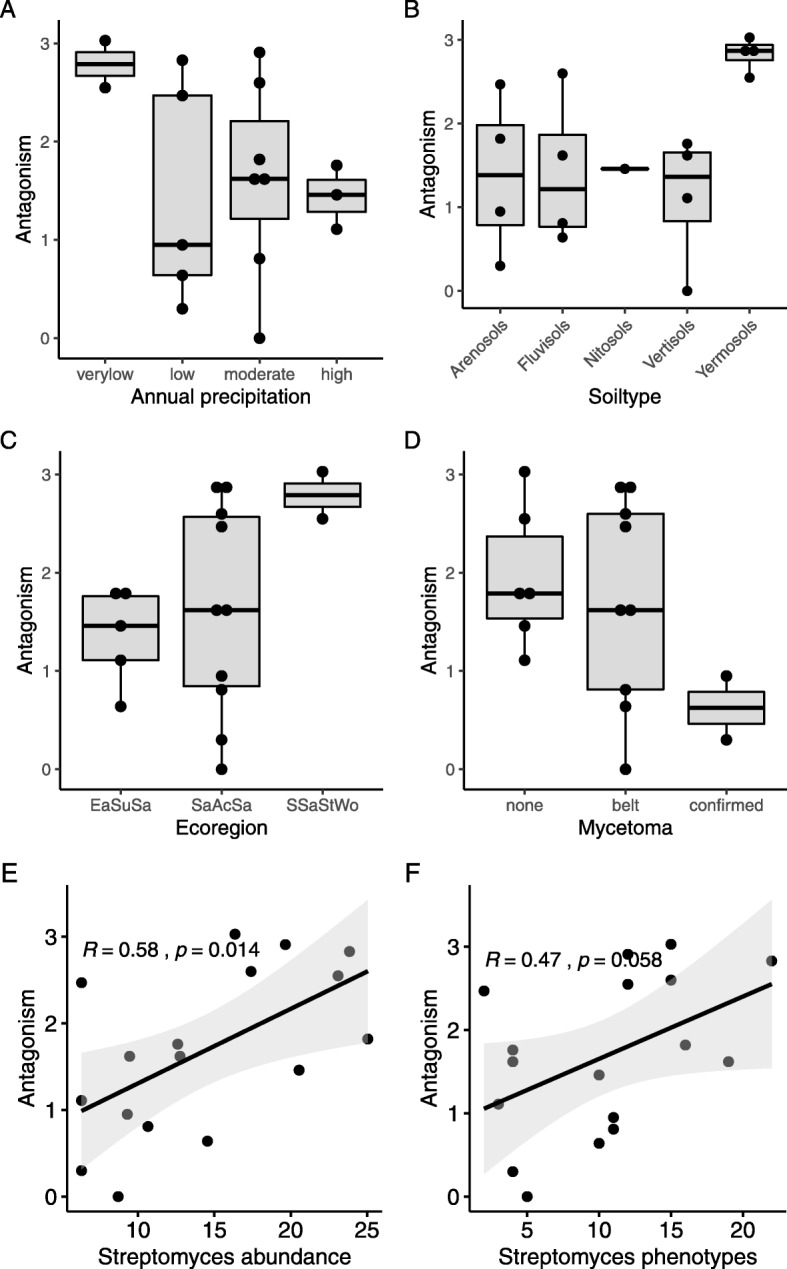
Antagonistic potential of soil streptomycetes. The level of antagonism was determined as the ratio between the inhibition zone against *S. sudanensis* and colony size of soil streptomycetes. Data are presented in dependency of annual precipitation (**a**), ecoregion (**b**), soil type (**c**), Mycetoma (**d**) *Streptomyces* abundance (**e**), and phenotypic diversity of *Streptomyces* isolates (**f**). Abundance corresponds to the mean value of colony forming units (× 10^5^ / g soil) on humic acid and ISP2 agar; Annual precipitation to precipitation levels 0–100 mm (very low), 101–400 mm (low), 401–600 mm (moderate) and 601–1000 mm (high); Ecoregion to East Sudanian savanna (EaSuSa), Sahelian Acacia savanna (SaAcSa) and South Saharan steppe and woodlands (SSaStWo), Mycetoma for the geographical origin of bacteria, from mycetoma belt, with confirmed, and without confirmed actinomycetoma. The Pearson correlation coefficients (R and *p*-value) are provided as measures of the strength of linear association between two variables

Antagonistic potential against *S. sudanensis* varied widely among the isolated soil streptomycetes. Of the 173 *Streptomyces* strains, 115 (66.5%) showed inhibitory effects against *S. sudanensis* (Additional file 5). Differences in antagonistic potential were related to the three different ecological regions (Fig. 3). Strains from the South Saharan steppe and woodlands ecoregion exhibited higher mean inhibition values (2.79 ± 0.24) compared to the East Sudanian savanna ecoregion (1.36 ± 0.22; *p* = 0.028) as well as compared to the Sahelian Acacia savanna ecoregion (1.79 ± 0.32; *p* = 0.025) (Fig. 3c, Additional file 4 and Additional file 5). Soils from areas with low annual precipitation, 70–200 mm per year (sites 7, 19), showed slightly higher inhibitory activities against *S. sudanensis* than sites with a higher precipitation (Fig. 3a). The level of antagonism correlated with the mean abundance of streptomycetes on humic acid and ISP2 media and, in particular, with the abundance of streptomycetes on humic acid medium (*p* = 0.002). This was illustrated by a positive correlation between the mean abundance of streptomycetes on humic acid and ISP2 media and their antagonistic activity (Fig. 3e; Pearson correlation R = 0.58, *p* = 0.014).

**Fig. 4:**
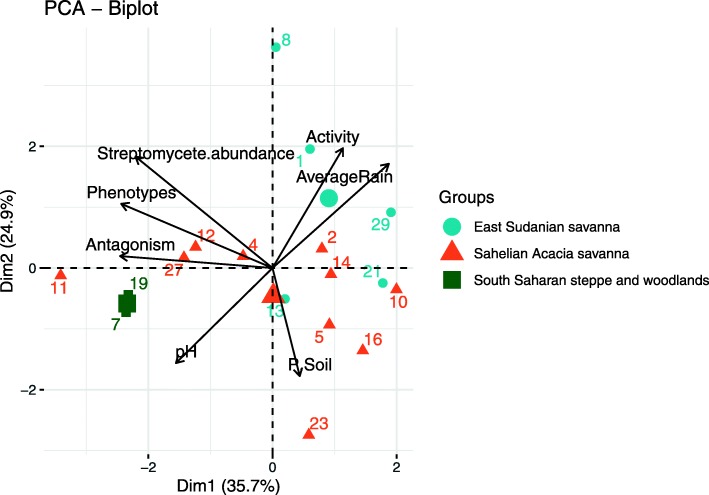
Principal coordinate analysis (PCoA) displaying *Streptomyces* isolate collections from three ecoregions. *Streptomyces* isolate collections of the three ecoregions in relation to the abundance of *Streptomyces* strains and *Streptomyces* phenotypes, level of antagonism against *S. sudanensis*, soil enzyme activity, pH, phosphorus and average rainfall. The numbers indicate the sites of isolation. Activity: soil enzyme activities; Abundance: number of streptomycetes colonies on HA and ISP2 agar; Antagonism: inhibitory activity against *Streptomyces sudanensis*

To sum up the main drivers of the *Streptomyces* abundance and inhibitory potential, a principal coordinate analysis was implemented (Fig. 4). The plot underlined the positive correlations between abundance and inhibitory potential of *Streptomyces* collections, negative correlations with precipitation, and visualized the grouping of the streptomycetes according to the three ecoregions.

## Discussion

The results of this study showed that: (1) site-specific parameters affected the abundance, composition and *S. sudanensis* inhibitory potential of cultivable soil *Streptomyces* community, (2) increased abundance and inhibitory potential of the streptomycete community were associated with low annual rainfall and Yermosol soil type, and (3) increased relative abundance and phenotypic diversity of *Streptomyces* isolates leads to an increased inhibitory potential against *S. sudanensis*.

Streptomycetes were successfully isolated from different Sudanese soils and about two third of the strains inhibited *S. sudanensis*. This is consistent with previous studies that indicated the potential of streptomycetes to inhibit other strains of the same genus [17, 18]. Comparing the mean inhibitory activity of isolates, site-dependent patterns related to the ecoregion-specific soil type and annual rainfall were detected. Associating the inhibitory streptomycetes from the three ecoregions including the mycetoma belt and outside it indicated the following conclusions: the inhibitory activity of soil streptomycetes from South Saharan steppe outside the mycetoma belt was significantly higher than that of bacteria from the Sahelian Acacia savanna within the mycetoma belt. Also, the inhibitory activity of soil streptomycetes from South Saharan steppe was higher than those from the East Sudanian savanna. In contrast, East Sudanian savanna and the Sahelian Acacia savanna showed no significant difference in inhibitory activities. This suggests local adaptation of streptomycetes [14, 19], and indicates that South Saharan steppe sites outside the mycetoma belt with Yermosol soil type are suitable for the search of *Streptomyces* bacteria producing antimicrobials against *S. sudanensis*.

Interestingly, strains isolated from sites where actinomycetoma occurs (Sahelian Acacia savanna) showed a comparably low inhibitory potential than those isolated from mycetoma free East Sudanian savanna, but the streptomycetes isolated from the likewise mycetoma free South Saharan steppe ecoregion showed a significantly higher average inhibitory potential. Also the inhibitory potential is particularly low at sites 10 and 14 where we have the confirmed sites of actinomycetoma [9]. Sudan, like many sub-Saharan African countries, has large expanses of diverse soils, expansive clay in the east-central area and sand dunes in the center with variable climatic conditions defining wide-ranging ecological areas [14]. Our data on *S. sudanensis* inhibitory bacteria contribute to the view that soil properties do affect the inhibitory potential of streptomycetes. This is in particular evident for sites 7 and 19 of the South Saharan steppe and woodlands with Yermosol soil type, which demonstrated higher antagonism to *S. sudanensis* compared to the East Sudanian savanna and Sahelian Acacia savanna ecoregions. Antibiotic inhibition and resistance as well as resource use efficiency are crucial for competitive interactions among *Streptomyces* spp., and in diverse soil communities, the most competitive strains inhibit the strains that are dependent on same resources, and tolerant against the antibiotics of the competitors [12]. Soil is a highly heterogeneous and spatially structured environment, and the microhabitats (pores) in the soil provide ecological niches to form different microbial consortia. Thus, the spatial soil structure is an important factor in the evolution and maintenance of bacterial traits including antibiotic production. It has been observed that antibiotic production is enhanced in a spatially structured habitat and suggested that particular soil types (those with greater physical structure) favor growth of antibiotic-producing microorganisms [20]. The Yermosol soils of the arid South Saharan steppe and woodlands sites have different characteristics than the other sites in the south, west, central or eastern Sudan (Table 1) and these site specific characteristics are reflected by the structure and activity of the cultivable *Streptomyces* community. This agrees with results derived from the arid and semi-arid soils in areas in Israel, which were related to specific environmental factors rather than to geographic distances and spatial distribution patterns [21]. The abundance and activity of streptomycetes were at their highest under low soil moisture, and it has been established that prolonged drought periods characteristically lead to an increase in the relative abundance and activity of Actinobacteria. For instance, our preliminary results from grassland soils under severe experimental drought suggests that cellulose decomposing *Streptomyces* species are enriched and do maintain their functional properties under low soil moisture (M. T. and T. R., unpublished data). Although the sand dunes in western Sudan (sites 10, 14 and 23) seem physically similar to those in the north (sites 7 and 19), the strains from the latter are more active than those from the sandy semi desert (sandy dunes) of the west Sudan. Here other factors such as annual rainfall may be the reason behind the presence of different types of *Streptomyces* species.

To assess the soil-borne human health risk in actinomycetoma areas, it is essential to know which microorganisms are present in the soils and what are the functions of these species. Actinomycetes isolated in the present study were identified and assessed for their interaction with *S. sudanensis*. It is apparent from Additional file 5 that isolates can be separated from one another based on partial 16S rRNA genes and some can be distinguished from known *Streptomyces* species as they form distinct phylogenetic lines in the *Streptomyces* 16S rRNA gene tree (Fig. 1). It is, therefore, proposed that these isolates can be recognized as new species, which require detailed phenotypic characterizations. 16S rRNA gene sequence has been the gold standard for classification of prokaryotic microorganisms, nevertheless, there is no consensus on the precise level of genetic difference that defines a species [22]. A 0.5 to 1% difference (99 to 99.5% similarity) is often used [23], corresponding to a difference of 5 to 15 bp in the whole 16S rRNA gene sequence [24].

Some specific sites revealed unique streptomycete 16S rRNA gene clusters, including site number 14 in the mycetoma belt where 3 out of 11 isolates were assigned to *S. djakartensis* (Fig. 3).*S. djakartensis* was strongly inhibitory against *S. sudanensis* whereas other strains from this site show low activity. This suggests that phylogenetic assignment can be related to inhibitory activity. By contrast, the *S. werraensis* phylotypes showed different levels of inhibitory activities, ranging from no inhibition to strong antagonism of *S. sudanensis,* which is in line with the observations in the global survey of *Streptomycetes* [12].

An interesting outcome of this study is the fact that some of our soil isolates are causal agents of actinomycetoma. However, none of the soil isolates were found to be closely related to *S. somaliensis* or *S. sudanensis,* the recognized causal agents of actinomycetoma [9, 25]. However, strains originated from cases of actinomycetoma (SD524, SD528 and SD552) from our previous unpublished study were found to have high similarity with current soil isolates. Strains 2A, 2B, 2D, 10C, 8E, 16E, 21A, 27A and 29B aligned along with SD552 in the subclade of *S. werraensis*. Strains in subclade 6 (5F, 12E, 27I) aligned with SD524; whereas, strain SD528 aligned with *S. griseostramineus* along with soil isolates 7B, 11E, 11G, 19H, 27E, 27F, 27 L. Moreover, *S. werraensis* is believed to be one of the causal agent of fissure scab, a new lesion type of potato in South Africa, which lead to serious yield losses of the local potato industry [26].

## Conclusions

Antagonism against *S. sudanensis* is widely expressed by soil streptomycetes in Sudanese soils. Our study identified that *S. sudanensis* inhibiting streptomycetes are enriched in particular in areas with low precipitation level, and that they are abundant in Yermosoils. Our data suggest that changes in the presence, diversity and traits of inhibitory *Streptomyces* bacteria may affect the abundance and virulence of *S. sudanensis*. The two sites where actinomycetoma occurs, showed a low number and diversity of antagonistic soil streptomycetes that, moreover, exhibited particularly low inhibitory potentials against *S. sudanensis*. Future work shall focus on the impact of the antagonistic soil bacteria on the soil *S. sudanensis* population, and investigate antimicrobials production by the strongest inhibitors of this human pathogen.

## Methods

### Soil sampling sites

**Fig. 5:**
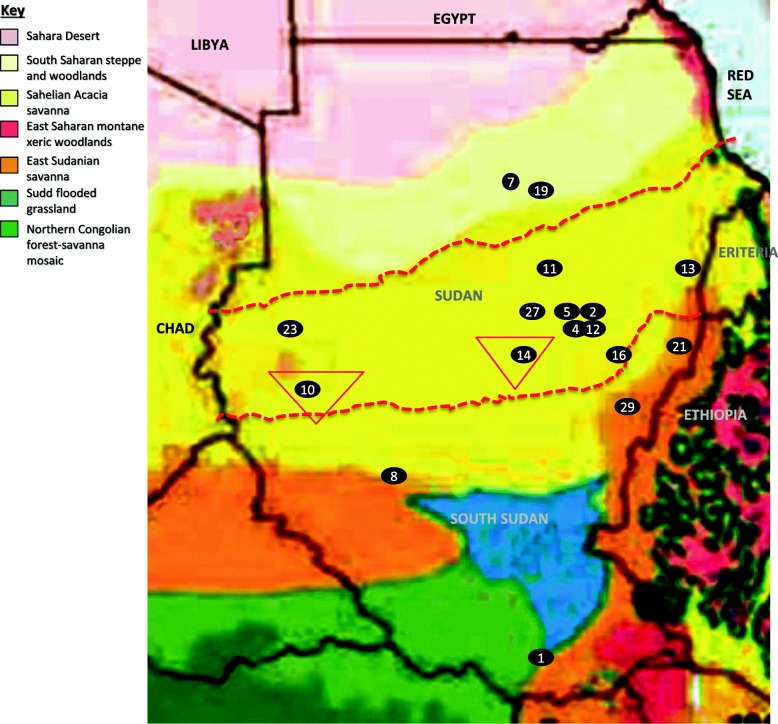
Terrestrial ecoregions of Sudan and South Sudan adapted from Burgess et al. [14]. The map shows *soil collection sites (black spots), the* mycetoma prevalence belt (thick broken red line) according to [27] and the confirmed *Streptomyces sudanensis* areas (inverted red triangles) from [9]. Key to sites: 1, Juba, Republic of South Sudan; 8, El Muglad, West Kordofan state; 10, Nyala, South Darfur state; 11, Soba, Khartoum state; 13, Kassala, Kassala state; 14, Umm Ruwaba, North Kordofan state; 16, Sennar, Sennar State; 21, Basonda, Al Gadarif state; 2, 4, 5 and 12, Hajj Abd Allah, Gazira state; 23, Al Fashir, North Darfur state; 7 and 19, Hussein Narti Northern state; 27, Ad Douiem, White Nile state; and 29, Ad Damazin, Blue Nile state. Written permission was obtained for the use of this figure from Island Press

Soil was collected from 17 sites in 12 states of Sudan and South Sudan during the dry season (January to March) in 2016 (Fig. 5). The sites lie within three different terrestrial ecoregions of Sudan and South Sudan according to Burgess et al. [14], namely the South Saharan steppe and woodlands, the Sahelian Acacia savanna and the East Sudanian savanna. In comparison to the South Saharan steppe and woodlands, the mean precipitation levels in the other two ecoregions were higher, but varied along the sampling sites. The East Sudanian savanna include sites in the southern Sudan and South Sudan with moderate to high rainfall (sites 1, 8, 21 and 29) whereas the Sahelian Acacia savanna includes sites in central, eastern and western Sudan with a low to moderate rainfall (sites 2, 4, 5, 10, 11, 12, 13, 14, 16, 23 and 27). In total, 10 g from five sampling points within each site were collected from the topsoil layer (0–10 cm depth) by using sterilized spatula, pooled, sieved and mixed well to form a composite sample for each site. Composite samples were transferred into sterile plastic bags, labeled, transported to laboratory and stored at 4 °C until further analyses.

### Soil physicochemical parameters

pH was measured with an electrode after shaking the soil for 1 hour in 0.01 M calcium chloride solution (1:2.5 w/v). Plant available P and K were extracted from fresh soils with double lactate (1:50 w/v, pH 3.6, 1.5 h [28];). After filtration of the suspension (Whatman Schleicher & Schuell 595 1/5 Ø 270 mm), the extracted P was colorimetrically quantified using the molybdenum blue method [29], while K was measured with an ion-selective electrode (perfectIONTM, Mettler Toledo, Gießen, Germany).

### Soil enzyme assays

Determination of the activities of five hydrolytic enzymes was based on the procedure of German et al. [30] using 4 methylumbelliferone (MUB)-coupled substrates. The substrates used in this study were 4-MUB-β-D-cellobioside, 4-MUB-β-D-glucoside, 4-MUB-β-D-xyloside, 4-MUB-N-acetyl-β-D-glucosaminide and 4-MUB-phosphate in order to estimate the activity of enzymes which are involved in carbon (β-glucosidase, cellobiohydrolase, xylosidase), nitrogen (N-acetylglucosaminidase), and phosphorus (phosphatase) acquisition. The final substrate concentrations in the assay were adapted in a pre-test ensuring that each enzyme was assayed under saturating conditions, in order to avoid an underestimation of enzyme activities [31]. Two grams of soil were pre-incubated with 400 μl of sterile water for 24 h at 4 °C. Soil suspensions were prepared by adding 0.8 g of soil to 50 ml sodium acetate buffer (50 mM, pH 5) and subsequent sonication for 5 min. Approximately 0.35 g of soil was dispersed into 50 ml of 50 mM Na-Acetate Buffer (pH 5) through sonication for 5 min. The soil suspensions were added to respective MUB-coupled substrates in a microtiter plate with eight technical replicates and incubated for 1 h at 25 ± 1 °C in the dark. Shortly before measurement, NaOH was added to all wells to enhance fluorescence of MUB, which was excited at 360 nm and measured at 465 nm using a TECAN Infinite F200 PRO plate reader (TECAN, Crailsheim, Germany). Fluorescence values in the assay and control wells were corrected with auto-fluorescence values of soil suspension and buffer, respectively. MUB standards (1.25 and 2.5 μM) dissolved in buffer and soil suspensions were used to determine emission and quench coefficients. Enzyme activities (nmol ˑ h^− 1^ ˑ g dry soil^− 1^) were calculated according to German et al. [30], whereby turnover rates (nmol ˑ h^− 1^) were related to the amount of dry soil.

### Isolation of *Streptomyces* from soil

High nitrogen content (HNC) medium (6% yeast extract, 0.05% SDS, 0.05% CaCl_2_ [pH 7.0]) [32] was used to facilitate extraction and isolation of streptomycetes. Soil (0.5 g dry weight) was added to liquid HNC medium and mixed well. The inoculated HNC medium was placed on a preheated shaker and rotated at 120 rpm and 42 °C for 1 h. Subsequently, the suspension was let to settle for 5 min and decanted to a clean Falcon tube. Samples were diluted (1:5; 1:10, 1:30) and 0.1 mL of each dilution as well as of the undiluted sample was evenly spread onto ISP2 agar and on humic acid agar (HA) plates using sterile Drigalski spatula. ISP2 agar [33] was supplemented with cycloheximide (50 mg/L), nystatin (40 mg/L) and nalidixic acid (54.9 mg/L) to inhibit bacterial and fungal contamination. A sterile filtered vitamin solution (1 mL/L - pH 7 - containing 12.5 μg folate, 12.5 μg biotin 250 μg *p*-aminobenzoic acid, 1.25 mg thiamine-HCl, 1.5 mg pantothenic acid, 1.25 mg riboflavin, 2.875 mg nicotinic acid and 125 μg vitamin B12) was added to enhance the growth of streptomycetes. Inoculated plates were incubated at 27 °C for up to 3 weeks. For purification, colonies showing streptomycetes-typical morphology were streaked on ISP2 agar. Pure cultures were stored at − 20 °C in sterile vials containing 20% glycerol until further analysis.

### Phylogenetic classification of *Streptomyces* isolates

PEG 200 (polyethylene glycol, Sigma-Aldrich) was used for the isolation of DNA from grown streptomycetes after modified Chomczynski and Rymaszewski method [34]. The solution was composed of 52 mL PEG 200, 39 mL distilled water, 2.95 mL 2 M KOH (pH 13.3–13.5). Before use, the solution was autoclaved and stored at 4 °C. For DNA extraction, 300 μL PEG solution, 1 glass bead and an inoculation loop with the bacterial colony were mixed. Bacterial cells were lysed by incubation for 15 min at RT, and subsequently the suspension was directly used for PCR amplification.

The 16S rDNA was amplified using universal primers 27F: 5′-AGAGTT TGA TCC TGG CTC AG-3′ and 1492R: 5′-GGT TAC CTT GTT ACG ACT T-3′ [35]. Amplification reactions were performed with Promega Green Mix (Promega) with the following thermal cycling conditions: initial denaturation at 94 °C for 5 min; 31 cycles at 95 °C for 30 s, 54 °C for 90 s and 72 °C for 120 s; and a final extension at 72 °C for 5 min. The amplification reaction was performed by Bio-Rad thermal cycler (MyCycler, Bio-Rad, USA) and the amplified products were examined by 1% agarose gel electrophoresis.

The Sanger sequencer ABI 3730XL 96-capillary DNA analyzer (Applied Biosystems) and SeqMan software (DNA star) were used to determine and assemble the gene sequences. The 16S rRNA gene sequences of 175 strains were aligned with published sequences by BLAST against the whole NCBI-nr database (http://www.ncbi.nlm.nih.gov/) and sequence relatedness was visualized using MEGA 7 software [16]. A phylogenetic tree was constructed using the neighbor-joining method in MEGA7 program, using the Kimura’s two-parameter model [36] with bootstrap values based on 1000 replications. All isolates, including isolates that could not be assigned up to the species level as well as strains that form individual clusters are listed in Additional file 5, together with details of their antagonistic potential, accession numbers and their 16S rRNA gene similarity values with related *Streptomyces* spp.

### Interaction assay

The inhibition of *S. sudanensis* was evaluated for each isolate using an agar-based bioassay. Fresh suspensions (0.1 mL) prepared from soil streptomycetes were placed on ISP2 agar plates, which was streaked before with *S. sudanensis* (DSM 41923). More than one *Streptomyces* isolate was cultured per plate. The inhibitory activities of soil streptomycetes against *S. sudanensis* were calculated as the ratio between the diameter of the inhibition zone and the antagonist colony diameter.

### Statistics

Data were analyzed using R (R Development Core Team 2008) and PAST (Version 3.14; Øyvind Hammer, Natural History Museum, University of Oslo, 1999–2016). One-way analysis of variance and Tukey post hoc test was used to evaluate if *Streptomyces* collections differ from each other. Permutational multivariate analysis of variance (PerMANOVA) was used to compare enzyme activity patterns of soils from the different sites, and Pearson correlation was used to estimate interdependency of variables.

## Supplementary information

**Additional file 1. **Soil enzymatic activity potential in dependency with annual precipitation levels. Principal component analysis reflects the relatedness of cellulose, beta-glucosidase, xylosidase, N-acetylglucosaminidase and phosphatase activities with annual rainfall. Enzyme activities were positively related to the level of precipitation according to PERMANOVA analysis (*p* = 0.033)

**Additional file 2.** Soil enzymatic activity potentials related to the level of annual precipitation and to soil type. Annual rainfall, precipitation levels 0–100 mm (very low), 101–400 mm (low), 401–600 mm (moderate) and 601–1000 mm (high).

**Additional file 3. **Isolation of *Streptomyces* species from soil using ISP2 (A) and humic acid agar (B). Examples of purified *Streptomyces* colonies (C) and inhibition of *Streptomyces sudanensis* growth by some soil streptomycetes (D).

**Additional file 4. **Characterization of *Streptomyces* collection at the level of 16S rRNA genes. Strain affiliation, antagonism against *Streptomyces sudanensis,* identity according to partial 16S rRNA gene sequence, percent identity to nucleotide sequence at GenBank, and accession number are given.

**Additional file 5. **Summary statistics of *Streptomyces* isolates from three ecoregions. The total sizes of culture collections from the three ecoregions, South Saharan steppe and woodlands, Sahelian Acacia savanna and East Sudanian savanna, and the antagonistic activities of the strains against *Streptomyces sudanensis.*

## Data Availability

The datasets used and/or analyzed during the current study are available from the corresponding author on reasonable request. The partial bacterial 16S rRNA gene sequences are deposited at NCBI under the accession numbers MF353938-MF353991 and MF356310-MF356365. Source organisms and the degree of homology to publicly available bacterial 16 S rRNA gene sequences are given in Additional file 4.

## Abbreviations

DSM: Deutsche Sammlung von Mikroorganismen- German Collection of Microorganisms
HA: Humic acid agar medium
HNC: High nitrogen content medium
ISP2: International Streptomyces Project Medium 2
