# Supplementary material for: Diversity and geographic distribution of soil streptomycetes with antagonistic potential against actinomycetoma-causing Streptomyces sudanensis in Sudan and South Sudan
Source: BMC Microbiol. 2020 Feb 12;20:33. doi: 10.1186/s12866-020-1717-y (PMC7017484; doi:10.1186/s12866-020-1717-y)
Supplement: Supplementary file 1 — Additional file 1. Soil enzymatic activity potential in dependency with annual precipitation levels. Principal component analysis reflects the relatedness of cellulose, beta-glucosidase, xylosidase, N-acetylglucosaminidase and phosphatase activities with annual rainfall. Enzyme activities were positively related to the level of precipitation according to PERMANOVA analysis (p = 0.033) [file 12866_2020_1717_MOESM1_ESM.docx]

**
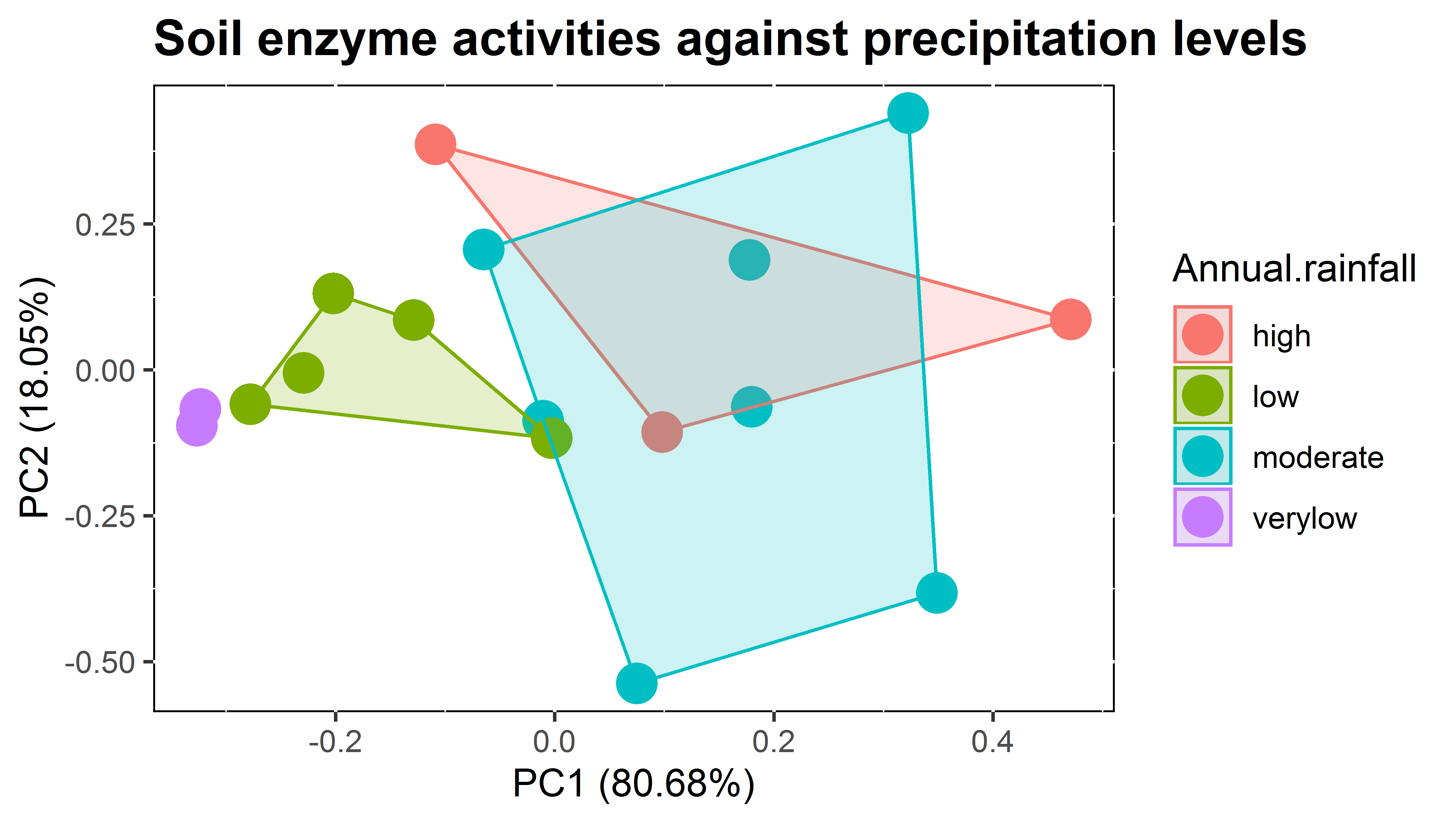
**

**Additional File 1** Soil enzymatic activity potential in dependency with annual precipitation levels. Principal component analysis reflects the relatedness of cellulose, beta-glucosidase, xylosidase, N-acetylglucosaminidase and phosphatase activities with annual rainfall. Enzyme activities were positively related to the level of precipitation according to PERMANOVA analysis (*p* = 0.033)
