# Supplementary material for: Diversity and geographic distribution of soil streptomycetes with antagonistic potential against actinomycetoma-causing Streptomyces sudanensis in Sudan and South Sudan
Source: BMC Microbiol. 2020 Feb 12;20:33. doi: 10.1186/s12866-020-1717-y (PMC7017484; doi:10.1186/s12866-020-1717-y)
Supplement: Supplementary file 2 — Additional file 2. Soil enzymatic activity potentials related to the level of annual precipitation and to soil type. Annual rainfall, precipitation levels 0–100 mm (very low), 101–400 mm (low), 401–600 mm (moderate) and 601–1000 mm (high). [file 12866_2020_1717_MOESM2_ESM.docx]

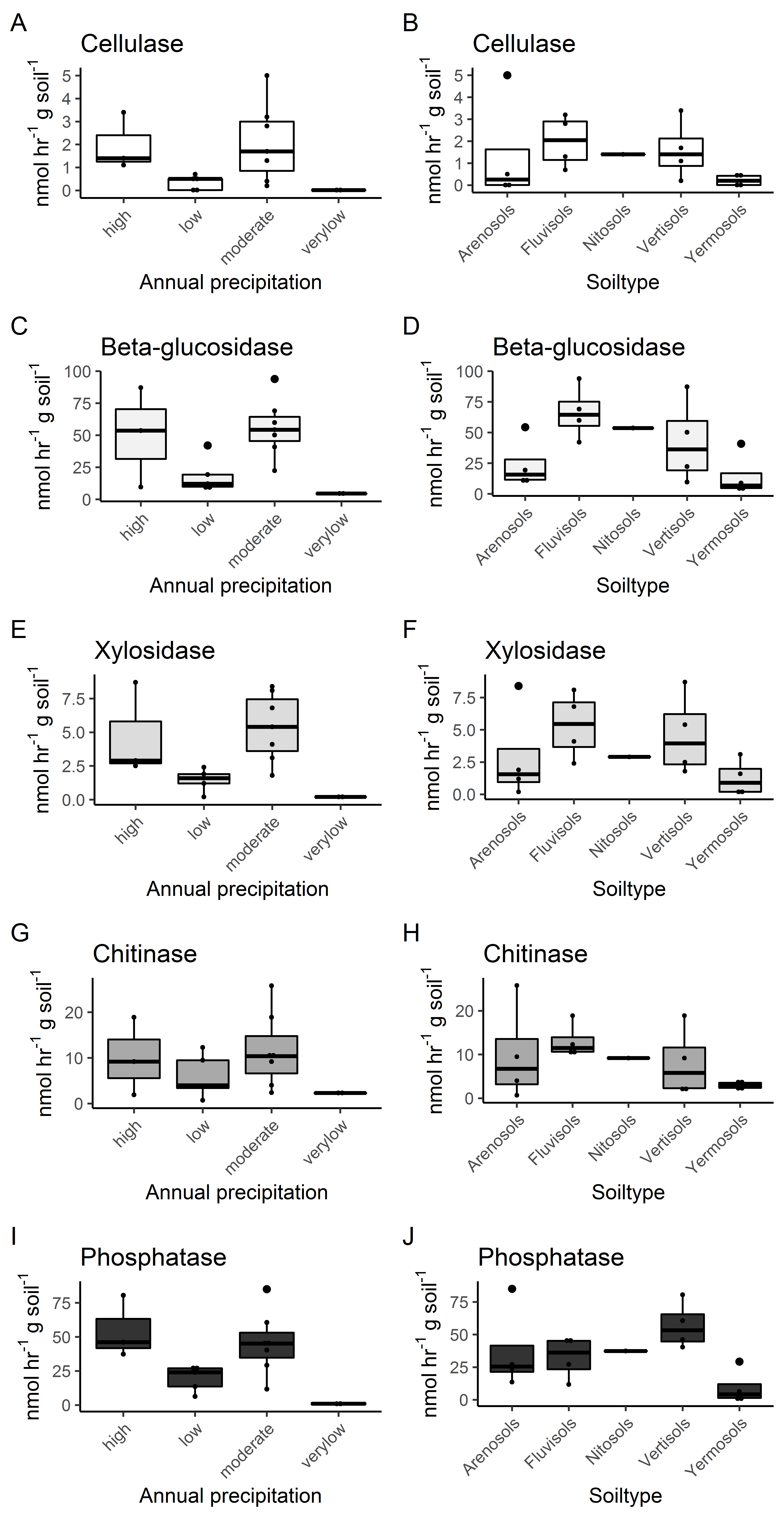


**Additional File 2** Soil enzymatic activity potentials related to the level of annual precipitation and to soil type. Annual rainfall, precipitation levels 0-100 mm (very low), 101-400 mm (low), 401-600 mm (moderate) and 601-1000 mm (high).
