# Supplementary material for: Diversity and geographic distribution of soil streptomycetes with antagonistic potential against actinomycetoma-causing Streptomyces sudanensis in Sudan and South Sudan
Source: BMC Microbiol. 2020 Feb 12;20:33. doi: 10.1186/s12866-020-1717-y (PMC7017484; doi:10.1186/s12866-020-1717-y)

**Additional File 3** Isolation of *Streptomyces* species from soil using ISP2 (A) and humic acid agar (B). Examples of purified *Streptomyces* colonies (C) and inhibition of *Streptomyces sudanensis* growth by some soil streptomycetes (D).


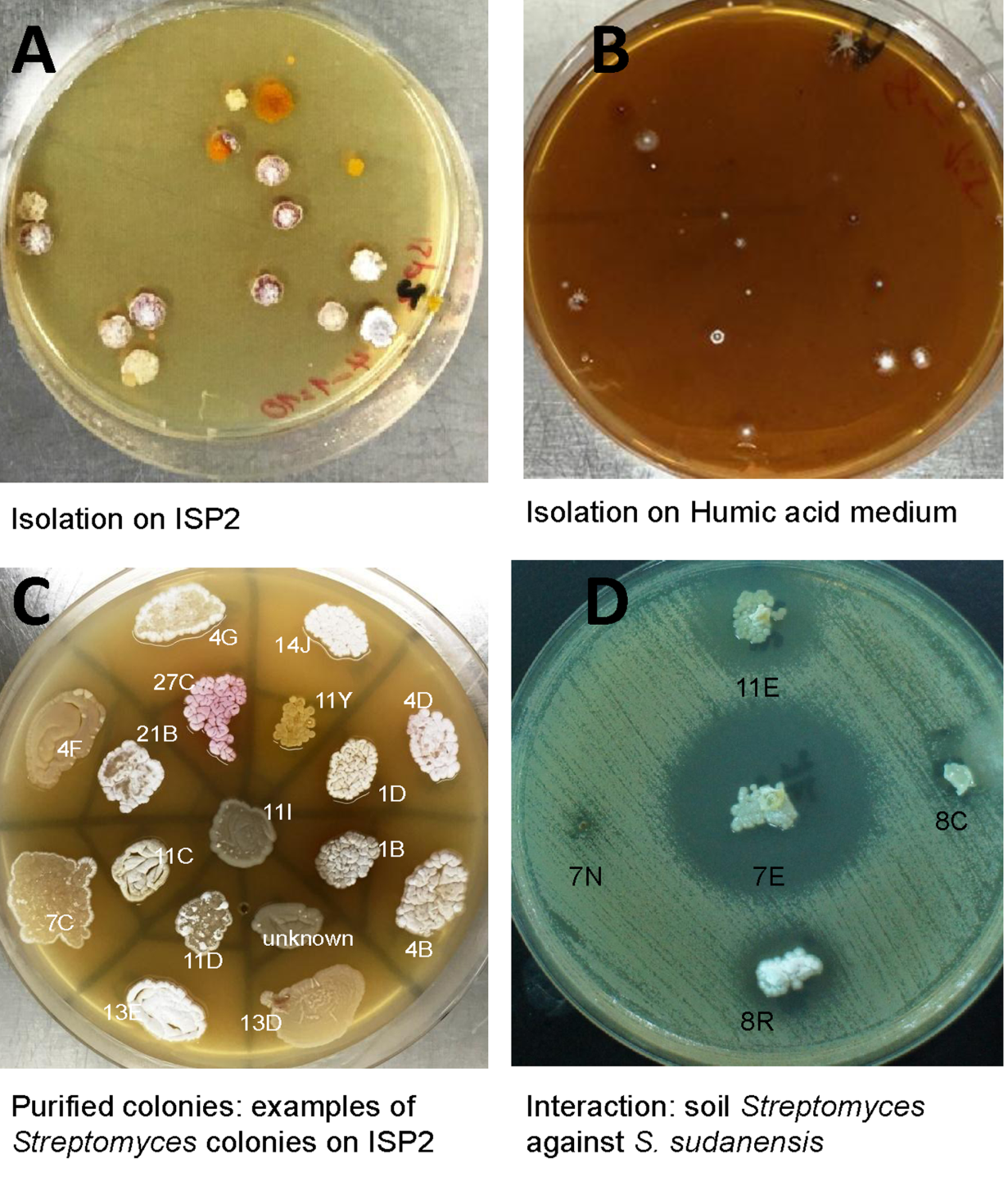

Supplement: Supplementary file 3 — Additional file 3. Isolation of Streptomyces species from soil using ISP2 (A) and humic acid agar (B). Examples of purified Streptomyces colonies (C) and inhibition of Streptomyces sudanensis growth by some soil streptomycetes (D). [file 12866_2020_1717_MOESM3_ESM.docx]
