# Supplementary material for: Diversity and geographic distribution of soil streptomycetes with antagonistic potential against actinomycetoma-causing Streptomyces sudanensis in Sudan and South Sudan
Source: BMC Microbiol. 2020 Feb 12;20:33. doi: 10.1186/s12866-020-1717-y (PMC7017484; doi:10.1186/s12866-020-1717-y)
Supplement: Supplementary file 4 — Additional file 4. Characterization of Streptomyces collection at the level of 16S rRNA genes. Strain affiliation, antagonism against Streptomyces sudanensis, identity according to partial 16S rRNA gene sequence, percent identity to nucleotide sequence at GenBank, and accession number are given. [file 12866_2020_1717_MOESM4_ESM.docx]

**Additional File 4** Characterization of *Streptomyces* collection at the level of 16S rRNA genes.

| **Running number** | **Strain code** | **Antagonism (clearance zone/ colony diameter)** | **Identification** | **% Nucleotide identity** | **Gb_ Accession** |
| --- | --- | --- | --- | --- | --- |
|  | 1A | 0.00 | *Streptomyces* sp. | - | NA |
|  | 1B | 1.67 | *Streptomyces* sp. | - | NA |
|  | 1C | 0.00 | *Streptomyces levis* | 100.00% with *S. levis* | MF353956 |
|  | 1D | 2.60 | *Streptomyces* sp. | - | NA |
|  | 1E | 0.00 | *Streptomyces* sp. | - | NA |
|  | 1F | 0.00 | *Streptomyces iakyrus* | 100.00% with *S. iakyrus* | MF353957 |
|  | 1G | 1.33 | *Streptomyces* sp. | - | NA |
|  | 1H | 2.50 | *Streptomyces* sp. | 97.94% with *S. misionensis* | MF356336 |
|  | 1I | 2.50 | *Streptomyces* sp. | - | NA |
|  | 1J | 4.00 | *Streptomyces hawaiiensis* | 99.64% with *S. hawaiiensis* | MF353958 |
|  | 2A | 1.33 | *Streptomyces werraensis* | 99.88% with *S. werraensis* | MF353970 |
|  | 2B | 1.71 | *Streptomyces werraensis* | 100% with *S. werraensis* | MF353971 |
|  | 2C | 2.00 | *Streptomyces* sp. | 98.34% with *S. enissocaesilis* and 98.34% with *S. rochei* | NA |
|  | 2D | 1.43 | *Streptomyces werraensis* | 99.58% with *S. werraensis* | MF353972 |
|  | 4A | 0.00 | *Streptomyces* sp. | - | NA |
|  | 4B | 3.50 | *Streptomyces prasinosporus* | 99.70% with *S. prasinosporus* | MF353973 |
|  | 4C | 1.33 | *Streptomyces* sp. | - | NA |
|  | 4D | 2.00 | *Streptomyces prasinosporus* | 99.90% with *S. prasinosporus* | MF353974 |
|  | 4E | 1.67 | *Streptomyces* sp. | - | NA |
|  | 4F | 2.20 | *Streptomyces variabilis* | 97.41% with *S. variabilis* | MF353975 |
|  | 4G | 2.75 | *Streptomyces rochei* | 100% with *S. enissocaesilis* and 100% with *S. rochei* | NA |
|  | 4H | 4.50 | *Streptomyces* sp. | 98.89% with *S. werraensis* | MF356344 |
|  | 4I | 0.00 | *Streptomyces rubiginosus* | 99.70% with *S. pseudogriseolus* | MF353976 |
|  | 4J | 0.00 | *Streptomyces* sp. | 99.62% with *S. griseoflavus* | MF356345 |
|  | 4K | 0.00 | *Streptomyces* sp. | - | NA |
|  | 4L | 2.50 | *Streptomyces prasinosporus* | 100% with *S. prasinosporus* | MF353977 |
|  | 4M | 0.00 | *Streptomyces* sp. | 97.73 with *S. werraensis* | MF356346 |
|  | 4N | 5.00 | *Streptomyces* sp. | - | NA |
|  | 4O | 0.00 | *Streptomyces* sp. | - | NA |
|  | 4P | 4.00 | *Streptomyces enissocaesilis* | 99.88%% with *S. enissocaesilis* and 99.88% with *S. rochei* | NA |
|  | 4Q | 0.00 | *Streptomyces* sp. | - | NA |
|  | 4R | 1.33 | *Streptomyces* sp. | - | NA |
|  | 4S | 1.50 | *Streptomyces prasinosporus* | 100.00% with *S. prasinosporus* | MF353978 |
|  | 5A | 1.40 | *Streptomyces* sp. | - | NA |
|  | 5B | 1.29 | *Streptomyces* sp. | - | NA |
|  | 5C | 1.50 | *Streptomyces* sp. | 99.70% with *S. atrovirens* | MF356347 |
|  | 5D | 0.00 | *Streptomyces flocculus* | 99.54% with *S. flocculus* | MF353979 |
|  | 5E | 1.25 | *Streptomyces rubiginosus* | 99.51% with *S. pseudogriseolus* | MF353980 |
|  | 5F | 3.50 | *Streptomyces* sp. | 99.65% with *S. althioticus* and 99.64% with *Streptomyces* sp. SD524 (EU544234) | MF356348 |
|  | 5G | 0.00 | *Streptomyces chilikensis* | 99.90% with *S. chilikensis* | MF353981 |
|  | 5H | 0.00 | *Streptomyces* sp. | -- | NA |
|  | 5I | 0.00 | *Streptomyces* sp. | 97.59% with *S. niveoruber* | MF356349 |
|  | 5J | 0.00 | *Streptomyces atrovirens* | 99.76% with *S. atrovirens* | MF353982 |
|  | 5K | 0.00 | *Streptomyces* sp. | - | NA |
|  | 7A | 0.00 | *Streptomyces* sp. | 97.00% with *S. griseostramineus* | MF356350 |
|  | 7B | 4.00 | *Streptomyces griseostramineus* | 99.89% with *S. griseostramineus*. 99.89% with *S. griseomycini* and 99.89% with *Streptomyces* sp. SD528 (EU544233) | MF353983 |
|  | 7C | 2.33 | *Streptomyces* sp. | 97.63% with *S. chromofuscus* | MF356351 |
|  | 7D | 6.25 | *Streptomyces* sp. | 99.33% with *S. levis* | MF356352 |
|  | 7E | 5.30 | *Streptomyces* sp. | 98.80% with *S. albaduncus* | MF356353 |
|  | 7F | 3.50 | *Streptomyces prasinosporus* | 99.90% with *S. prasinosporus* | MF353984 |
|  | 7G | 3.00 | *Streptomyces* sp. | 98.76% with *S. minutiscleroticus* | MF356354 |
|  | 7H | 2.50 | *Streptomyces leeuwenhoekii* | 99.77% with *S. leeuwenhoekii* | MF353985 |
|  | 7I | 2.50 | *Streptomyces* sp. | 99.08% with *S. albaduncus* | MF356355 |
|  | 7J | 2.50 | *Streptomyces* sp. | 98.72% with *S. vinaceusdrappus*. 99.88%% with *S. enissocaesilis* and 99.88% with *S. rochei* | NA |
|  | 7K | 0.00 | *Streptomyces enissocaesilis* | 100% with *S. enissocaesilis* and 100% with *S. roche*i | MF353986 |
|  | 7L | 2.50 | *Streptomyces* sp. | - | NA |
|  | 7M | 0.00 | *Streptomyces* sp. | 97.32% with *S. niveoruber* | MF356357 |
|  | 7N | 5.00 | *Streptomyces* sp. | 99.18% with *S. chromofuscus* | MF356358 |
|  | 7O | 6.00 | *Streptomyces* sp. | 99.52% with *S. werraensis* | MF356359 |
|  | 8A | 3.00 | *Streptomyces albogriseolus* | 99.79% with *S. albogriseolus* | MF353987 |
|  | 8B | 0.00 | *Streptomyces albogriseolus* | 100% with *S. albogriseolus* | MF353988 |
|  | 8C | 4.00 | *Streptomyces* sp. | 97.94% with *S. kebangsaanensis* | MF356360 |
|  | 8D | 0.00 | *Streptomyces variabilis* | 99.90% with *S. variabilis* | MF353989 |
|  | 8E | 0.00 | *Streptomyces werraensis* | 99.70% with *S. werraensis* | MF353990 |
|  | 8F | 1.33 | *Streptomyces* sp. | 99.13% with *S. albogriseolus* | MF356361 |
|  | 8G | 2.33 | *Streptomyces* sp. | 99.51% with *S. pomoeae* | MF356362 |
|  | 8H | 0.00 | *Streptomyces albogriseolus* | 99.77% with *S. albogriseolus* | MF353991 |
|  | 8I | 0.00 | *Streptomyces* sp. | - | NA |
|  | 8J | 1.67 | *Streptomyces* sp. | - | NA |
|  | 8K | 2.29 | *Streptomyces* sp. | - | NA |
|  | 8L | 3.33 | *Streptomyces* sp. | 98.32% with *S. misionensis* | MF356363 |
|  | 8M | 2.22 | *Streptomyces* sp. | - | NA |
|  | 8N | 1.33 | *Streptomyces* sp. | 99.37% with *S. lomondensis* and 99.12% with *S. luteogriseus* | MF356364 |
|  | 8O | 4.00 | *Streptomyces* sp. | - | NA |
|  | 8P | 1.33 | *Streptomyces* sp. | **-** | NA |
|  | 8R | 4.17 | *Streptomyces* sp. | 97.29% with *S. fumigatiscleroticus* | MF356365 |
|  | 10A | 0.00 | *Streptomyces* sp. | - | NA |
|  | 10B | 0.00 | *Streptomyces enissocaesilis* | 99.30% with *S. enissocaesilis* and 99.30 with *S. rochei* | MF353938 |
|  | 10C | 1.20 | *Streptomyces werraensis* | 100% with *S. werraensis* | MF353939 |
|  | 10D | 0.00 | *Streptomyces enissocaesilis* | 99.41% with *S. enissocaesilis* and 99.41% with *S. rochei* | MF353940 |
|  | 11A | 0.00 | *Streptomyces* sp. | - | NA |
|  | 11B | 0.00 | *Streptomyces* sp. | - | NA |
|  | 11C | 5.00 | *Streptomyces* sp. | - | NA |
|  | 11D | 3.67 | *Streptomyces glaucus* | 99.77% with *S. glaucus* | MF353941 |
|  | 11E | 5.00 | *Streptomyces griseostramineus* | 100% with *S. griseostramineus* | MF353942 |
|  | 11F | 3.50 | *Streptomyces* sp. | - | NA |
|  | 11G | 2.33 | *Streptomyces griseostramineus* | 100% with *S. griseostramineus* | MF353943 |
|  | 11H | 2.50 | *Streptomyces* sp. | 96.36% with *S. werraensis* | MF356310 |
|  | 11I | 4.00 | *Streptomyces* sp. | 99.57% with *S. lomondensis* and 99.35% with *S. luteogriseus* | MF356311 |
|  | 11J | 1.33 | *Streptomyces fimbriatus* | 100 % with *S. fimbriatus* | MF353944 |
|  | 11K | 1.92 | *Streptomyces* sp. | - | NA |
|  | 11L | 2.50 | *Streptomyces* sp. | - | NA |
|  | 11M | 4.29 | *Streptomyces* sp. | - | NA |
|  | 11N | 3.50 | *Streptomyces* sp. | 96.68% with *S. prasinosporus* | NA |
|  | 11O | 0.00 | *Streptomyces* sp. | - | NA |
|  | 11P | 4.00 | *Streptomyces* sp. | - | NA |
|  | 11Q | 3.30 | *Streptomyces* sp. | - | NA |
|  | 11R | 3.0 | *Streptomyces* sp. | - | NA |
|  | 11S | 0.0 | *Streptomyces* sp. | 97.89% with *S. werraensis* | MF356312 |
|  | 11T | 6.0 | *Streptomyces* sp. | -- | NA |
|  | 11U | 3.3 | *Streptomyces* sp. | - | NA |
|  | 11W | 3.00 | *Streptomyces* sp. | - | NA |
|  | 12A | 2.33 | *Streptomyces* sp. | - | NA |
|  | 12B | 4.57 | *Streptomyces* sp. | - | NA |
|  | 12C | 5.00 | *Streptomyces* sp. | 97.22% with *S. niveoruber* | MF356313 |
|  | 12D | 1.20 | *Streptomyces* sp. | 96.34% with *S. rubiginosus* | MF356314 |
|  | 12E | 1.50 | *Streptomyces* sp. | 98.13% with *S. althioticus* and 98.67% with *Strptomyces* sp. SD524 (EU544234) | MF356315 |
|  | 12F | 3.00 | *Streptomyces* sp. | - | NA |
|  | 12G | 2.33 | *Streptomyces* sp. | 97.16% with *S. glaucus* | MF356316 |
|  | 12H | 0.00 | *Streptomyces* sp. | - | NA |
|  | 12I | 2.00 | *Streptomyces chromofuscus* | 98.98% with *S. chromofuscus* | MF353945 |
|  | 12J | 0.00 | *Streptomyces* sp. | - | NA |
|  | 12K | 5.00 | *Streptomyces* sp. | 97.34% with *S. chromofuscus* | MF356317 |
|  | 12L | 0.00 | *Streptomyces* sp. | 96.84% with *S. chromofuscus* | MF356318 |
|  | 12M | 4.00 | *Streptomyces* sp. | - | NA |
|  | 12Mwh | 4.00 | *Streptomyces* sp. | 97.89% with *S. glaucus* | MF356319 |
|  | 12Y | 4.00 | *Streptomyces* sp. | - | NA |
|  | 13A | 0 | *Streptomyces* sp. | - | NA |
|  | 13B | 0 | *Streptomyces* sp. | 96.78% with *S. deserti* | MF356320 |
|  | 13C | 2.33 | *Streptomyces* sp. | - | NA |
|  | 13D | 0 | *Streptomyces* sp. | 97.27% with *S. fumanus* | MF356321 |
|  | 13E | 0 | *Streptomyces* sp. | 97.44% with S. atrovirens | MF353946 |
|  | 13G | 2.10 | *Streptomyces* sp. | - | NA |
|  | 13H | 0 | *Streptomyces* sp. | - | NA |
|  | 13I | 0 | *Streptomyces* sp. | 94.53% with *S. glaucus* | MF356322 |
|  | 13J | 1.33 | *Streptomyces* sp. | - | NA |
|  | 14A | 2.00 | *Streptomyces* sp. | - | NA |
|  | 14B | 0.00 | *Streptomyces* sp. | 97.80% with *S. fragilis* | MF356324 |
|  | 14C | 0.00 | *Streptomyces* sp. | 96.83% with *S. leeuwenhoekii* | MF356325 |
|  | 14D | 0.00 | *Streptomyces* sp. | 96. 32% with *S. leeuwenhoekii* | MF356326 |
|  | 14E | 0.00 | *Streptomyces djakartensis* | 100% with *S. djakartensis* | MF353947 |
|  | 14F | 0.00 | *Streptomyces* sp. | - | NA |
|  | 14G | 0.00 | *Streptomyces* sp. | 96.52% with *S. hawaiiensis* | MF356327 |
|  | 14H | 3.00 | *Streptomyces* sp. | 98.88% with *S. minutiscleroticus* | MF356328 |
|  | 14I | 2.50 | *Streptomyces* sp. | 96.30% with *S. aurantiogriseus* | MF356329 |
|  | 14J | 3.00 | *Streptomyces djakartensis* | 100% with *S. djakartensis* | MF353948 |
|  | 14K | 0.00 | *Streptomyces djakartensis* | 100% with *S. djakartensis* | MF353949 |
|  | 16A | 0.00 | *Streptomyces prasinosporus* | 99.90% with *S. prasinosporus* | MF353950 |
|  | 16B | 0.00 | *Streptomyces enissocaesilis* | 99.52% with *S. enissocaesilis* and 99.52% with *S. rochei* | MF353951 |
|  | 16C | 0.00 | *Streptomyces* sp. | - | NA |
|  | 16E | 0.00 | *Streptomyces werraensis* | 100% with *S. werraensis* | MF353952 |
|  | 19A | 2.50 | *Streptomyces* sp. | 94.79% with *S. malachitofuscus* | MF356331 |
|  | 19B | 1.30 | *Streptomyces* sp. | 98.54% with *S. minutiscleroticus* | MF353953 |
|  | 19C | 0.00 | *Streptomyces* sp. | - | NA |
|  | 19D | 1.25 | *Streptomyces* sp. | 99.55% with *S. werraensis* | MF356332 |
|  | 19E | 2.00 | *Streptomyces* sp. | 94.91% with *S. werraensis* | MF356333 |
|  | 19F | 0.00 | *Streptomyces* sp. | - | NA |
|  | 19G | 2.75 | *Streptomyces* sp. | - | NA |
|  | 19H | 6.00 | *Streptomyces griseostramineus* | 99.77% with *S. griseostramineus* 99.77% with *Streptomyces* sp. SD528 (EU544233) | MF353954 |
|  | 19I | 6.67 | *Streptomyces werraensis* | 100% with *S. werraensis* | MF353955 |
|  | 19J | 3.00 | *Streptomyces* sp. | 99.33% with *S. werraensis* | MF356334 |
|  | 19K | 3.50 | *Streptomyces* sp. | 95.56% with *S. thermocarboxydovorans* | MF356335 |
|  | 19L | 1.67 | *Streptomyces* sp. | - | NA |
|  | 21A | 0.00 | *Streptomyces werraensis* | 99.60% with *S. werraensis* | MF353959 |
|  | 21B | 3.33 | *Streptomyces prasinosporus* | 99.30% with *S. prasinosporus* | MF353960 |
|  | 21C | 0.00 | *Streptomyces* sp. | 93.63% with *S. tendae* | NA |
|  | 23A | 3.33 | *Streptomyces* sp. | - | NA |
|  | 23B | 1.60 | *Streptomyces fragilis* | 99.68% with *S. fragilis* | MF353961 |
|  | 27A | 3.00 | *Streptomyces werraensis* | 99.50% with *S. werraensis* | MF353962 |
|  | 27B | 3.33 | *Streptomyces* sp. | 98.81% with *S. niveoruber* | MF356338 |
|  | 27C | 3.33 | *Streptomyces* sp. | 96.66% with *S. minutiscleroticus* | MF356339 |
|  | 27D | 2.50 | *Streptomyces* sp. | 99.61% with *S. tuirus* | MF356340 |
|  | 27E | 3.33 | *Streptomyces griseostramineus* | 99.77% with *S. griseostramineus* | MF353963 |
|  | 27F | 2.50 | *Streptomyces griseostramineus* | 100% with *S. griseostramineus*. 100% with *S. griseomycini* and 100% with *Streptomyces* sp. SD528 (EU544233) | MF353964 |
|  | 27G | 3.00 | *Streptomyces prasinosporus* | 99.71% with *S. prasinosporus* | MF353965 |
|  | 27H | 2.67 | *Streptomyces* sp. | 98.63% with *S. lomondensis* | MF356341 |
|  | 27I | 5.00 | *Streptomyces* sp. | 99.31% with *S. althioticus* and 99.31% with *Strptomyces* sp. SD524 (EU544234) | MF356342 |
|  | 27J | 0.00 | *Streptomyces* sp. | 99.43% with *S. tuirus* | MF356343 |
|  | 27L | 3.33 | *Streptomyces griseostramineus* | 99.78% with *S. griseostramineus* and 100% with *Streptomyces* sp. SD528 (EU544233) | MF353966 |
|  | 29A | 2.00 | *Streptomyces* sp. | - | NA |
|  | 29B | 2.29 | *Streptomyces werraensis* | 99.88% with *S. werraensis* | MF353967 |
|  | 29C | 2.75 | *Streptomyces cinnabarinus* | 98.48% with *S. cinnabarinus* | MF353968 |
|  | 29D | 0.00 | *Streptomyces werraensis* | 99.63% with *S. werraensis* | MF353969 |

Strain affiliation, antagonism against *Streptomyces sudanensis,* identity according to partial 16S rRNA gene sequence, percent identity to nucleotide sequence at GenBank, and accession number are given.
