# Supplementary material for: Diversity and geographic distribution of soil streptomycetes with antagonistic potential against actinomycetoma-causing Streptomyces sudanensis in Sudan and South Sudan
Source: BMC Microbiol. 2020 Feb 12;20:33. doi: 10.1186/s12866-020-1717-y (PMC7017484; doi:10.1186/s12866-020-1717-y)
Supplement: Supplementary file 5 — Additional file 5. Summary statistics of Streptomyces isolates from three ecoregions. The total sizes of culture collections from the three ecoregions, South Saharan steppe and woodlands, Sahelian Acacia savanna and East Sudanian savanna, and the antagonistic activities of the strains against Streptomyces sudanensis. [file 12866_2020_1717_MOESM5_ESM.docx]

**Additional File 5** Summary statistics of *Streptomyces* isolates from three ecoregions.

|  | **East Sudanian savanna** | **Sahelian Acacia savanna** | **South Saharan steppe and woodlands** |
| --- | --- | --- | --- |
| **Number of strains** | 34 | 112 | 27 |
| **Number of active strains** | 22 | 71 | 22 |
| **Proportion of active strains** | 64.7 | 63.4 | 81.5 |
| **Minimum inhibitory effect** | 0 | 0 | 0 |
| **Maximum inhibitory effect** | 4.17 | 6 | 6.67 |
| **Sum of inhibitory effect** | 55.97 | 201.64 | 76.02 |
| **Mean inhibitory effect** | 1.65 | 1.80 | 2.82 |
| **Std. error** | 0.248 | 0.157 | 0.391 |

The total sizes of culture collections from the three ecoregions, South Saharan steppe and woodlands, Sahelian Acacia savanna and East Sudanian savanna, and the antagonistic activities of the strains against *Streptomyces sudanensis*
